# Higher aggrecan 1-F21 epitope concentration in synovial fluid early after anterior cruciate ligament injury is associated with worse knee cartilage quality assessed by gadolinium enhanced magnetic resonance imaging 20 years later

**DOI:** 10.1186/s12891-020-03819-9

**Published:** 2020-12-01

**Authors:** Paul Neuman, Staffan Larsson, L. Stefan Lohmander, André Struglics

**Affiliations:** 1 Orthopedics, Department of Clinical Sciences Malmö, Faculty of Medicine, Lund University, Lund, Sweden; 2 Orthopedics, Department of Clinical Sciences Lund, Faculty of Medicine, Lund University, Lund, Sweden

**Keywords:** ACL injury, Aggrecan, Biomarkers, Synovial fluid, dGEMRIC

## Abstract

**Background:**

To investigate if cartilage related biomarkers in synovial fluid are associated with knee cartilage status 20 years after an anterior cruciate ligament (ACL) injury.

**Methods:**

We studied 25 patients with a complete ACL rupture without subsequent ACL reconstruction or radiographic knee OA. All had a delayed gadolinium-enhanced magnetic resonance imaging of cartilage (dGEMRIC) 20 years after the ACL injury, using the T1 transverse relaxation time in the presence of gadolinium (T1Gd) which estimates the concentration of glycosaminoglycans in hyaline cartilage. Synovial fluid samples were aspirated acutely (between 0 and 18 days) and during 1 to 5 follow up visits between 0.5 and 7.5 years after injury. We quantified synovial fluid concentrations of aggrecan (epitopes 1-F21 and ARGS), cartilage oligomeric matrix protein, matrix metalloproteinase-3 and tissue inhibitor of metalloproteinase-1 by immunoassays, and sulfated glycosaminoglycans by Alcian blue precipitation. Western blot was used for qualitative analyses of aggrecan fragments in synovial fluid and cartilage samples.

**Results:**

Western blot indicated that the 1-F21 epitope was located within the chondroitin sulfate 2 region of aggrecan. Linear regression analyses (adjusted for age, sex, body mass index and time between injury and sampling) showed that acute higher synovial fluid 1-F21-aggrecan concentrations were associated with shorter T1Gd values 20 years after injury, i.e. inferior cartilage quality (standardized effects between − 0.67 and − 1.0). No other statistically significant association was found between molecular biomarkers and T1Gd values.

**Conclusion:**

Higher acute synovial fluid 1-F21-aggrecan concentrations in ACL injured patients, who managed to cope without ACL reconstruction and were without radiographic knee OA, were associated with inferior knee cartilage quality assessed by dGEMRIC 20 years after injury.

**Supplementary Information:**

The online version contains supplementary material available at 10.1186/s12891-020-03819-9.

## Background

Post-traumatic osteoarthritis (OA) is common after an anterior cruciate ligament (ACL) injury and is manifested by radiographic structural knee joint changes with osteophytes and decreased cartilage height, and with patients experiencing knee pain and stiffness [1–6]. Concomitant acute traumatic knee cartilage injuries are very common in ACL injured knees [7]. The mechanical damage is usually evidenced by superficial cartilage fibrillation and sometimes also with visible cracks down to the subchondral bone, and bone marrow lesions are present in almost every magnetic resonance imaging (MRI) after an acute ACL injury [8, 9]. Even if there is no visual damage to the cartilage surfaces at the time of arthroscopy there may be micro-damage to cartilage matrix and cell death especially in the superficial regions [10]. The ACL injury with cartilage damage triggers an immediate inflammatory response which acts in combination with an abnormal long-term mechanical loading of the injured knee believed to generate post-traumatic OA [11–13] .

We lack means to diagnose and treat early microscopic joint changes in cartilage; radiography is limited by its insensitivity in detecting these early joint changes, and they are not visible until years after disease onset when the cartilage might be beyond repair [14, 15]. Different molecular markers or combinations of biomarkers in synovial fluid, serum and urine have been suggested to be useful as prognostic OA-markers [16–22]. Altered turnover and loss of cartilage sulfated glycosaminoglycans (sGAG) is a recognized and important early event of the development of OA [23]. The delayed gadolinium-enhanced MRI of cartilage (dGEMRIC) is a non-invasive quantitative MRI technique that reflects the content of highly negatively charged macromolecules, such as sGAG, in the cartilage [24]. A strong correlation between dGEMRIC estimated cartilage sGAG content and histological scores has been found [25]. The dGEMRIC technique and study protocol have been validated [26], and clinically relevant associations between the dGEMRIC and risk factors for OA have been presented [27, 28]. The dGEMRIC technique has also proved to have a prognostic value for OA development [29–31].

Studies of associations between molecular biomarkers and MRI cartilage findings have been called for [32]. Only a couple of studies on association between synovial fluid molecular biomarkers and MRI cartilage findings 3 to 5 years after an ACL injury have been published [33, 34], and studies with longer follow-up time are lacking.

The aim of the present study was to examine if the concentration of molecular biomarkers in synovial fluid taken 0 to 7.5 years after ACL-injury were associated with knee cartilage quality assessed by dGEMRIC 20 years later.

## Methods

### Subjects and visits

**Fig. 1:**
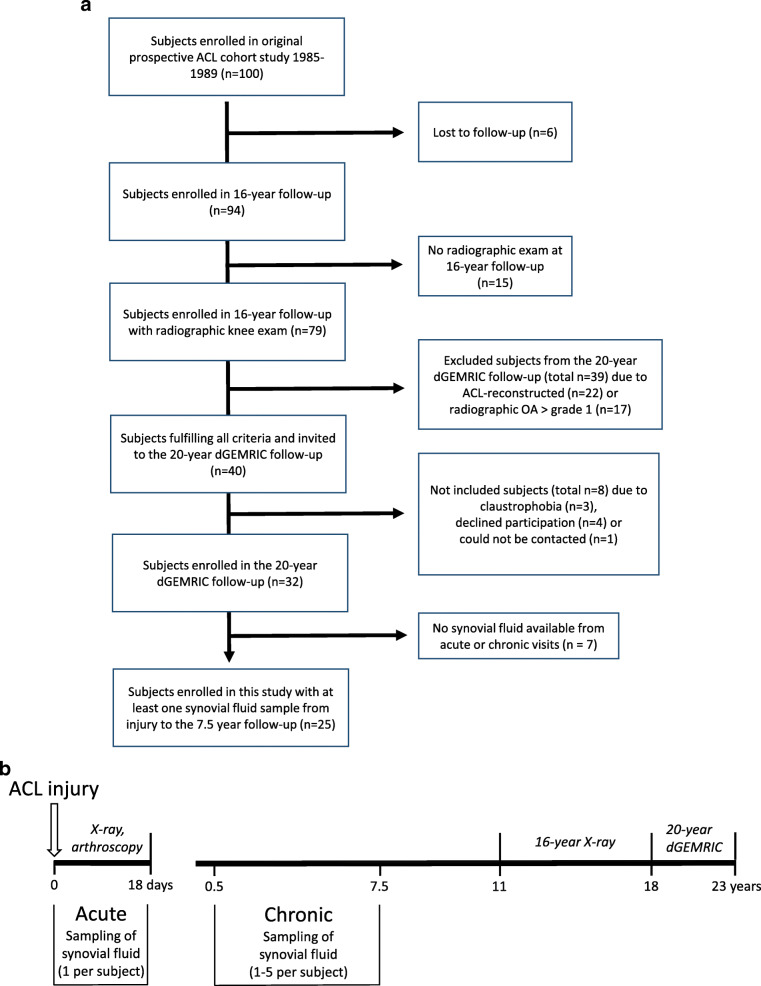
**a** Flow diagram of study subjects. **b** Timeline showing synovial fluid sampling and imaging and arthroscopic acquisitions. The 16-year x-ray examinations were done between 11 and 18 years after the ACL injury, while the 20-year dGEMRIC assessments were done 18 to 23 years after injury

Patients were from a well characterized cohort of 100 consecutive ACL-injured subjects prospectively recruited at the Lund University Hospital between 1985 and 1989 [35]. All 100 subjects had a complete ACL tear and were within 18 days after initial trauma assessed by arthroscopy and x-ray with no significant signs of pre-existing knee OA (Fig. 1a and b). The participants were treated with early physiotherapeutic knee rehabilitation without primary ACL reconstruction. Synovial fluid was collected early after injury (called acute visit; 0 to 18 days) and prospectively at 1 to 5 visits during the following 7.5 years (Fig. 1b). For another study with the purpose to examine the association between knee cartilage quality and knee function, 32 subjects without ACL reconstruction or radiographic signs of OA at the 16-year follow-up (described below) were examined with dGEMRIC 20 years after their ACL injury [36]. Since the dGEMRIC method is reliant on the presence of joint cartilage, only subjects having Osteoarthritis Research Society International (OARSI, [37]) atlas grades of ≤1 were included in the study. Twenty-five of the 32 subjects examined with dGEMRIC had one or more available synovial fluid sample aspirated following their injury and were included in this study (Fig. 1a and b, Table 1).

**Table 1:** Characteristics of the study subjects with dGEMRIC examination at the 20 years follow-up and available acute and/or chronic synovial fluid samples

	^a^Both dGEMRIC and SF samples, n	Time after injury to SF sampling	Age at injury mean (SD)	Men, %	BMI at injury, mean (SD)	BMI 20 years post injury, mean (SD)
Total study group	25	0 days to 7.5 years	24.5 (6.2)	52	23.6 (3.0)	25.3 (3.5)
	**Subjects with SF samples, n**	**Time after injury**				
Acute samples	20	0–18 days (median 6 days)				
Chronic samples	22	0.5–7.5 years (median 4 years)				
	4	0.5–1.5 years				
	17	1.5–2.5 years				
	12	2.5–3.5 years				
	11	3.5–4.5 years				
	10	4.5–5.5 years				
	3	5.5–6.5 years				
	1	6.5–7.5 years				

^a^ 25 subjects with any kind of SF-samples (i.e. acute and/or one or more chronic samples): 3 subjects had only acute samples, 5 subjects had only chronic samples and 17 subjects had both acute and chronic samples. Delayed gadolinium enhanced MRI of cartilage (dGEMRIC) examination: mean = 20.6 years (range = 18 to 23 years) after injury. *SF* Synovial fluid, *SD* Standard deviation.

### Radiography at the 16 year follow up

Radiographs at the 16 year (range 11–18 years) follow up were obtained in standardized standing anteroposterior knee position with both knees in 20 degrees of flexion and weight bearing on a tilt table; a fluoroscopically positioned x-ray beam was used to optimize medial tibial plateau alignment. The radiographs were independently read by two observers blinded to clinical details. Joint space narrowing (JSN) and osteophytes were graded independently on frontal images on a 4-point scale (range 0–3, 0 = no evidence of JSN or bony change) according to the OARSI atlas [14, 15, 37]. The interrater reliability (kappa statistic) was Κ = 0.78 for JSN and Κ = 0.52 for osteophytes [38].

### Synovial fluid sampling

Twenty-five subjects were included in this study with any kind of synovial fluid samples, i.e. either from first and/or following visit(s) as follows: 20 subjects had their synovial fluid aspirated at the acute visit within 18 days (median 6 days) after injury, and 22 subjects had their synovial fluids collected at between one and five visits during the subsequent 7.5 years of follow-up (median 4 years); these synovial fluids are called chronic samples (Fig. 1b, Table 1). The subjects visited the orthopedic outpatient ward only for study purposes [35, 38]. All synovial fluids were collected without joint lavage, and the samples were centrifuged at 3000×g for 10 min in room temperature and supernatants were stored at − 80 °C.

### Molecular marker analyses in synovial fluid

sGAG, in synovial fluid mainly chondroitin and keratan sulfate (CS and KS), was quantified by Alcian Blue precipitation [39]. Two different aggrecan epitopes were quantified using immunoassays and the monoclonal antibodies (mAb) 1-F21 and OA-1. According to previous publications, mAb 1-F21 is suggested to recognize a protein sequence within or close to the KS region of aggrecan [18, 40]. mAb OA-1 recognizes the ARGS neoepitope generated by aggrecanase cleavage at the TEGE^392^/^393^ARGS site in the interglobular domain of aggrecan [41]. Cartilage oligomeric matrix protein (COMP) was quantified using a commercial assay from AnaMar AB/IDS (cat. no. AN-14-1006-71); the AnaMar COMP-epitope has not been published. Matrix metalloproteinase-3 (MMP-3) and tissue inhibitor of metalloproteinase-1 (TIMP-1) were quantified using monoclonal and polyclonal antibodies; the MMP-3 immuno-assay recognizes both the pro- and active form of the protease and the complex with TIMP; the TIMP-1 immuno-assay detects only free TIMP-1 [42–44]. Data on ARGS-aggrecan was generated for this study, all other biomarker data were available from previous studies on the described ACL cohort [45, 46].

The ratio MMP-3/TIMP-1 was used to investigate differences in these biomarkers alone or as a ratio between the enzyme and its inhibitor. We further investigated the ratios of sGAG/COMP, ARGS-aggrecan/COMP and 1-F21 aggrecan/COMP as biomarkers; ratios like these have been suggested to minimize the influence of varying amounts of obtainable synovial fluid [47].

### Assessment with dGEMRIC at the 20 year follow up

Subjects were investigated with dGEMRIC on average 20.6 years (range between 18 and 23 years) after the ACL injury (Fig. 1b, Table 1). Briefly, Gd-DTPA^2−^ (Magnevist®, Schering AG, Berlin, Germany) was injected intravenously at a dose of 0.3 mmol/kg body weight. To optimize the uptake of Gd-DTPA^2−^ into the cartilage, subjects exercised by walking up and down the stairs for approximately ten minutes, starting 5 minutes after injection. Two hours after injection, post-contrast imaging of the cartilage was performed using a standard 1.5 T MRI system with a dedicated knee coil (Magnetom Vision; Siemens Medical Solutions, Erlangen, Germany). Central parts of the weight-bearing lateral and medial femoral cartilage were identified, and quantitative relaxation time calculations were performed in a 3 mm thick sagittal slice on each condyle, using sets of six turbo inversion recovery images with different inversion times: TR = 2000 ms, TE = 15 ms, FoV 120 × 120 mm^2^, matrix = 256 × 256, TI = 50, 100, 200, 400, 800 and 1600 ms. A full-thickness region of interest (ROI) in the cartilage was examined. T1Gd was calculated using the mean signal intensity from each ROI [48], and the dGEMRIC images were analyzed and ROIs were drawn using the MATLAB-based Mokkula software [26]. An orthopaedic surgeon performed the ROI measurements. All MRI data was available from a previous study [36].

### Western blot of aggrecan

Aggrecan fragments from synovial fluid (pooled from 47 subjects with knee OA or knee injury) were purified by mini-preparations of cesium-chloride density-gradient centrifugation in absence or presence of guanidinium chloride, collecting the associative A1 and dissociative D1 fractions, as described [49]. Purified aggrecan (i.e. A1D1 fraction prepared from pooled knee cartilage from ten subjects with OA) was in vitro digested using aggrecanase-1 (ADAMTS-4, a disintegrin and metalloproteinase with thrombospondin motifs-4) or MMP-3 as described [50]. The samples were deglycosylated and separated by SDS-PAGE on 3–8% Tris-acetate mini-gels and transferred to PVDF-membranes [39]. For the immune-reaction we used antibodies against aggrecan G1-domain (Affinity BioReagents no. PA1–1747, polyclonal IgG diluted 1:400), 1-F21 aggrecan epitope (IgG monoclonal antibody diluted 1:75000), ARGS-aggrecan epitope (IgG monoclonal neoepitope antibody OA-1 diluted to 5.3 μg/ml) and chondroitin sulfate clone 3B3 (Seikagaku no. 270789 IgM monoclonal antibody against chondroitinase treated chondroitin 6-sulfate diluted to 0.33 μg/ml). Secondary antibodies were peroxidase-conjugated horse anti-mouse IgG (CST no. 7076S diluted to 10 ng/ml), goat anti-mouse IgM (Sigma no. 8786 diluted to 10 ng/ml) and goat anti rabbit IgG (KPL no. 074–1516 diluted to 13 ng/ml). The immunobands were visualized using Pierce ECL Plus Western Blotting Substrate (no. 32132) and film (Amersham Hyperfilm ECL) or luminescence image analyser Bio-Rad ChemiDoc MP.

### Statistical analysis

Associations between the molecular biomarkers and dGEMRIC T1Gd values were investigated using linear regression models with adjustments for age at injury, sex, body mass index at dGEMRIC examination and time between injury and biomarker sampling. Results from crude (without adjustments) linear regression analyses are presented as a supplement (Table S1). Mann-Whitney tests were used for comparison of biomarker values between acute and chronic subject groups. For correlation analysis Spearman’s rank (r_S_) was used. For subjects with more than one chronic sample, the average biomarker concentration and the average time after injury were used in the linear regression model. The dGEMRIC values were normally distributed. Biomarker data were log10 transformed to obtain normal distribution. To be able to compare effect sizes between biomarkers, we report standardized effects from the linear regression analyses. The reported effects estimate how many standard deviations the dependent variable (dGEMRIC) will change per standard deviation increase in the predictor variable (biomarker concentration). All tests were 2-tailed and *P* ≤ 0.05 was considered statistically significant. The statistical analysis was performed with SPSS 24.0 for Windows software package.

## Results

### dGEMRIC (T1Gd) and synovial fluid biomarker values

The mean (standard deviation, SD) T1Gd dGEMRIC values at 20 years post injury for the 25 subjects was 397 ms (53) for the medial femoral cartilage, 431 ms (81) for the lateral femoral cartilage and 414 ms (58) for the medial and lateral femoral cartilage. For all biomarkers measured in synovial fluid, the concentrations were higher in the acute samples compared to chronic samples (Table 2).

**Table 2:** Concentration of biomarkers, expressed as median values (25th and 75th percentiles), in acute and chronic samples

Biomarkers	Acute samples		Chronic samples		^**1**^***P-***values
	Concentration	N	Concentration	N	
sGAG, μg/ml	181.8 (116.8, 323,8)	16	58.1 (42.8, 75,1)	21	< 0.001
1-F21 agcan, μg/ml	679.0 (413.4, 873.6)	16	117.0 (85.6, 168.3)	14	< 0.001
ARGS agcan, nM	11.5 (6.8, 21.1)	18	1.5 (1.1, 2.1)	22	< 0.001
COMP, μg/ml	180.0 (146.5, 214.5)	13	57.0 (52.3, 73.0)	12	< 0.001
MMP-3, nM	37.5 (21.4, 56.8)	18	5.2 (1.5, 8.5)	17	< 0.001
TIMP-1, nM	52.5 (43.1, 67.8)	18	7.3 (5.7, 9.3)	17	< 0.001
sGAG/COMP	1.08 (0.7, 1.6)	11	0.9 (0.8, 1.1)	12	0.295
1-F21 agcan/COMP	4.9 (1.7, 5.7)	11	1.5 (1.2, 2.5)	9	0.038
ARGS agcan/COMP	0.07 (0.04, 0.10)	12	0.02 (0.02, 0.03)	12	0.002
MMP-3/TIMP-1	0.7 (0.4, 1.1)	18	0.6 (0.3, 1.0)	17	0.642

^1)^ Statistical analyses using Mann Whitney.

*sGAG* Sulfated glycosaminoglycans, *1-F21 agcan* 1-F21 epitope of aggrecan, *ARGS agcan* ARGS neoepitope of aggrecan, *COMP* Cartilage oligomeric matrix protein, *MMP-3* Matrix metalloproteinase 3, *TIMP-1* Tissue inhibitor of metalloproteinase 1.

### Associations between synovial fluid biomarkers and dGEMRIC at 20 years

**Fig. 2:**
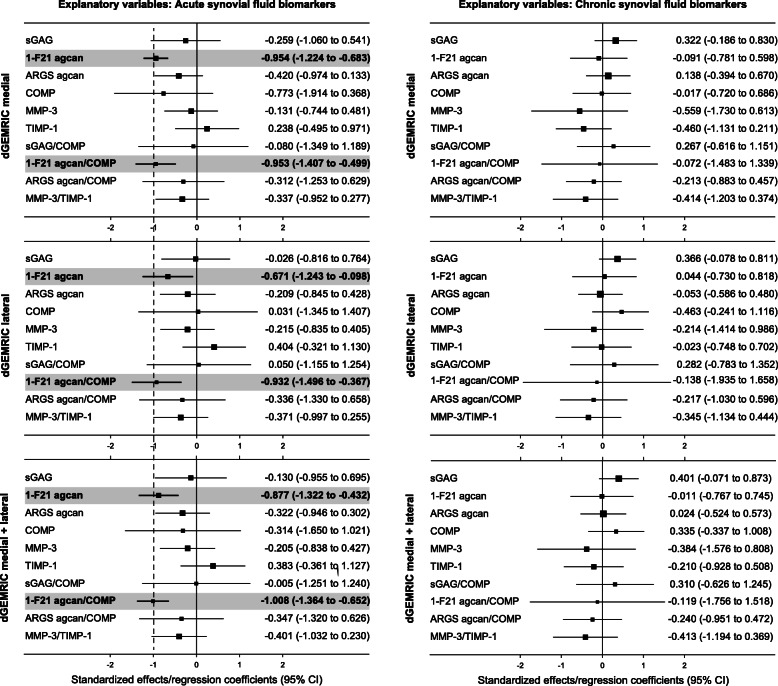
Adjusted linear regression analyses between molecular biomarkers and dGEMRIC. Molecular biomarkers in acute and chronic synovial fluid samples were used as prognostic variables for cartilage quality assessed by dGEMRIC 20 years post ACL injury. *Squares:* mean effect with size being proportional to number of available biomarker data. *Grey area:* highlights statistical significance with an alpha level of 0.05. *Standardized effect:* the estimate of the average change in dGEMRIC T1Gd (expressed as standard deviation) that corresponds to a 1 standard deviation change in the prognostic factor. 1-F21 agcan = 1-F21 epitope of aggrecan, ARGS agcan = ARGS neoepitope of aggrecan, COMP = cartilage oligomeric matrix protein, MMP-3 = matrix metalloproteinase 3, sGAG = sulfated glycosaminoglycans, TIMP-1 = tissue inhibitor of metalloproteinase 1. dGEMRIC medial + lateral = the sum of medial and lateral dGEMRIC values divided by 2

Of all investigated biomarkers, the only statistically significant associations found were between dGEMRIC and 1-F21 aggrecan and 1-F21 aggrecan/COMP ratio in the acute samples (Fig. 2). These biomarker values were inversely associated with T1Gd values in the medial, lateral and combined compartments (Fig. 2). The standardized effect sizes ranged from − 0.67 to − 1.0, and were similar between 1-F21 aggrecan alone or as a ratio of 1-F21 aggrecan/COMP. Crude linear regression analyses between molecular biomarkers and dGEMRIC showed similar associations as the adjusted analyses (Supplementary Table S1).

### Investigation of aggrecan assay specificity

**Fig. 3:**
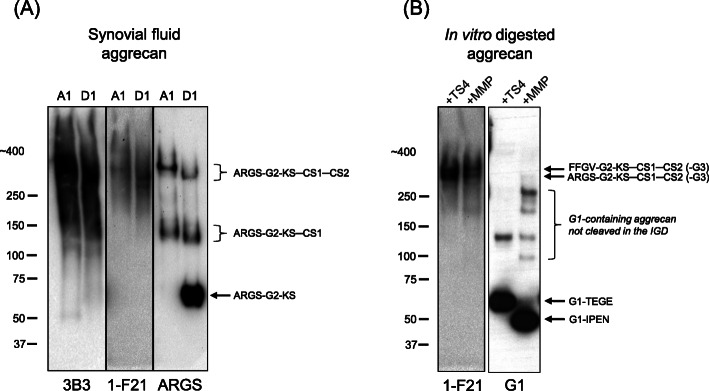
Western blot of synovial fluid and cartilage samples. **a** Synovial fluid A1 and D1 samples on membranes probed with antibodies against 6-sulfated chondroitin sulfate stubs (3B3), aggrecan epitope 1-F21 and ARGS-aggrecan. **b** ADAMTS-4 or MMP-3 in vitro digested cartilage A1D1 aggrecan samples on membranes probed with antibodies against aggrecan epitope 1-F21 and G1-domain of aggrecan. The position of Mw markers (left side) and the immunobands are indicated. The images are from different experiments showing representative signals. The original images from full size blotted gels are shown in Fig. S2. Keratan sulfate region (KS), chondroitin sulfate region (CS) and globular domains (G1, G2 and G3) are illustrated in Fig. 4. One to three μg sGAG was loaded per well. IGD = interglobular domain

There was a positive correlation between the aggrecan markers (1-F21 aggrecan, sGAG and ARGS-aggrecan) detected in the acute samples (r_S_ = between 0.697 and 0.789, *p* ≤ 0.006, *n* = 14–16; Fig. S1). Since only 1-F21 aggrecan of the three different aggrecan assays showed associations with subsequent cartilage quality, we investigated what type of aggrecan and proteoglycans the different quantitative aggrecan and proteoglycan assays detected in synovial fluid. In Western blots we used the same aggrecan antibodies as in the immunoassays (i.e. against ARGS-aggrecan and 1-F21 aggrecan) and as a control for Alcian Blue detected proteoglycans we used the 3B3 antibody. Samples used in these experiments were two different density-gradient centrifuge fractions (A1 and D1) of aggrecan purified from pooled synovial fluid. The result showed clear differences in the type of aggrecan fragments detected by the antibodies in synovial fluid (Fig. 3a). The ARGS-aggrecan antibody (mAb OA-1) detected three distinct protein fragments of aggrecan approximated to be ARGS-CS2, ARGS-CS1 and ARGS-KS. The 3B3 antibody detected the widest spectrum of aggrecan species, including fragments of the sizes of ARGS-CS2 and ARGS-CS1, but showed no, or very week reactivity against fragments around 64 kDa where ARGS-KS migrates. The 1-F21 antibody detected only high molecular weight species of sizes above 170 kDa, thus likely detecting the ARGS-CS2 species but not the ARGS-CS1 and ARGS-KS species (Fig. 3a).

**Fig. 4:**
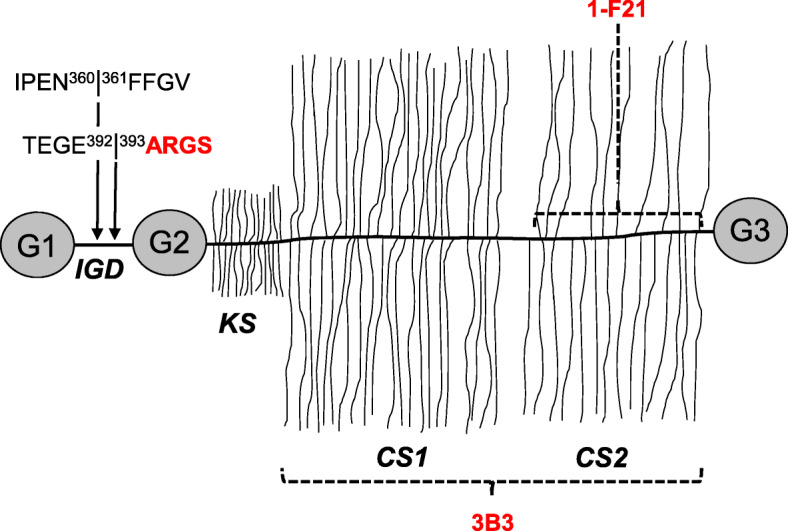
Schematic figure of aggrecan showing MMP (IPEN/FFGV) and aggrecanase (TEGE/ARGS) cleavage sites in the inter-globular domain (IGD). The amino acid numberings are based on the full-length human aggrecan amino acid sequence starting with the N-terminus ^1^MTTL and finishing with the C-terminus STAH^2415^ (NCBI accession no. P16112). The positions for recognition of 3B3 and aggrecan 1-F21 antibodies are shown by dashed lines. IGD = interglobular domain; KS = keratan sulfate region; CS = chondroitin sulfate region; G = globular domains

To further determine the location of the 1-F21 epitope, we made Western blots using samples of aggrecan which had been in vitro digested with ADAMTS-4 or MMP-3. The 1-F21 antibody detected high molecular aggrecan fragments of sizes corresponding to ARGS-CS2 and FFGV-CS2 in ADAMTS-4 or MMP-3 digested material, respectively (Fig. 3b). However, no reactivity was noted against the corresponding G1-TEGE and G1-IPEN fragments, or against ARGS-CS1 that is present in the ADAMTS-4 digested aggrecan sample (Fig. 3b). These results suggest that the 1-F21 epitope is located within the CS2 region of aggrecan (Fig. 4).

## Discussion

This study presents a long-term follow-up of an ACL-injury cohort where patients were treated with knee rehabilitation without ACL reconstruction and were without definite radiographic signs of radiographic OA 16 years after their injury. We found that in this patient group higher acute synovial fluid concentrations of large aggrecan fragments detected with the 1-F21 antibody were associated with lower T1Gd values measured by dGEMRIC 20 years later. None of the other investigated biomarkers measured acutely after injury or up to 7.5 years after injury were associated with dGEMRIC T1Gd at the follow up. Similar findings have been observed in rheumatoid arthritis, where subjects with destructive disease (that required joint replacement) had higher initial levels of 1-F21 aggrecan compared to subjects with non-destructive disease when evaluated up to 12 years later [47]. In accordance with previous studies evaluating knee injured subjects [27, 30, 31, 36] a slightly higher dGEMRIC value in the lateral than in the medial femoral cartilage was found also in this study. Medial and lateral dGEMRIC values in this study were not statistically different from control values in healthy uninjured subjects, indicating a still rather well preserved knee cartilage [36].

Using an ex vivo biomechanical cartilage injury model culturing explants in the presence of inflammatory cytokines, Wang et al. showed that large size aggrecan fragments were released from the injured cartilage momentarily and during the first 14 days [51]. Based on a similar cartilage explant model exposing the cartilage for cyclic loading, Orozco et al. showed a decrease in aggrecan concentration and presence of chondrocyte death around the cartilage cracks, which was not observed in the intact cartilage [52]. The same authors suggested that the early decrease of aggrecan in cartilage extracellular matrix following injury and subsequent tissue loading, without the addition of inflammatory drive, might be caused by the release of aggrecan through the damaged cartilage surface into the synovial cavity by high pressure fluid outflow. The cartilage leakage of structural proteins such as aggrecan into the synovial fluid is most likely dependent on the amount of compression and the shear forces on the joint surfaces at the trauma situation, but also on the quality of the affected cartilage. High quality knee cartilage of well-trained athletes is densely packed with proteoglycans, and higher synovial fluid concentrations of proteoglycans were found after an ACL injury in well-trained athletes compared to levels in less well-trained individuals with ACL injured knees [53]. However, in the patients from this cohort we found no association between the measured molecular biomarkers or T1Gd values and their rather uniform activity levels (data not shown).

Previous reports have suggested that the 1-F21 epitope resides within or close to the KS-region of aggrecan [40]. However, since neither the N-terminal fragments G1-TEGE and G1-IPEN, nor ARGS-KS-CS1 or the shorter ARGS-KS fragments were detected by the 1-F21 antibody in the Western blots, the position of the 1-F21 epitope is further distal and most likely resides within the CS2 region (Fig. 4).

Using the same assays as herein for the detection of aggrecan fragments in the synovial fluid we have shown that the concentration of 1-F21 aggrecan, ARGS-aggrecan and sGAG were increased directly after a knee injury [18, 20, 46, 54]. This increase is most likely caused by the knee trauma and subsequent inflammation as a part of the repair mechanism in the joints during the acute phase after injury [11]. From the Western blot investigation in this study it is evident that there are differences in which aggrecan fragments these three aggrecan assays detect. While the ARGS-aggrecan assay detects specific aggrecanase generated ARGS-fragments, the sGAG and 1-F21 assays detect a variety of similar broad range large aggrecan fragments, concordant with the strong correlation between the sGAG and 1-F21 biomarkers [18]. Although there was a strong positive correlation between the aggrecan markers for the acute samples in this study, only 1-F21 aggrecan was associated with dGEMRIC values.

There are limitations in this study. Although the study design planned for repeated sampling of synovial fluid from the injured knee over several years, we do not have a complete set of data from every subject (Table 1). The study cohort is a selected subgroup that managed to cope well with their ACL injury without ACL reconstruction and had no radiographic knee OA at long-term follow-up (i.e. OARSI atlas grades ≤1), and the results may thus not be generalizable to all ACL injured subjects. On the other hand, the selection of investigated patients could be an important factor to explain our results in this study. These ACL-injured subjects had few subsequent knee injuries that would blur the association between the magnitude of the first traumatic cartilage injury and dGEMRIC values 20 years later. Other knee injury studies are more variable regarding inclusion, sampling time, age of subjects and highly variable knee pathologies and surgeries which might influence the results from these cohorts [18, 20, 55].

A study showed that cartilage pre-contrast T1 and thickness are sources of variation in dGEMRIC indicating that well-trained elite runners with a thicker deep knee cartilage than sedentary volunteers achieve a higher dGEMRIC value (ms) just because of a thicker cartilage and not due to differences in cartilage structure [56]. This might be a limitation with the dGEMRIC method but is probably of less importance in our studied cohort which had a uniform low to medium high activity level.

## Conclusion

In conclusion, higher synovial fluid concentrations of large aggrecan fragments detected by the 1-F21 antibody early after ACL injury were associated with worse knee cartilage quality estimated by dGEMRIC 20 years later. High synovial fluid concentrations of large sized aggrecan fragments in acutely ACL injured knees may reflect the magnitude of the acute concomitant knee cartilage trauma, associated with later joint cartilage quality.

## Supplementary Information

**Additional file 1: Supplementary Table S1**: Crude linear regression analyses between molecular biomarkers and dGEMRIC,

**Additional file 2: Supplementary Figure S1**: Correlation between the aggrecan biomarkers 1-F21 aggrecan, sGAG and ARGS-aggrecan

**Additional file 3: Supplementary Figure S2**: Western blot of synovial and cartilage samples; the figure shows uncropped original sized images used in Fig. 3

## Data Availability

The datasets used and/or analysed during the current study are available from the corresponding author on reasonable request.

## Abbreviations

OA: Osteoarthritis
ACL: Anterior cruciate ligament
MRI: Magnetic resonance imaging
sGAG: Sulfated glycosaminoglycans
dGEMRIC: Delayed gadolinium-enhanced MRI of cartilage
OARSI: Osteoarthritis Research Society International
JSN: Joint space narrowing
CS: Chondroitin sulfate
KS: Keratan sulfate
mAb: Monoclonal antibody
COMP: Cartilage oligomeric matrix protein
MMP-3: Matrix metalloproteinase-3
TIMP-1: Tissue inhibitor of metalloproteinase-1
ROI: Region of interest
ADAMTS-4: A disintegrin and metalloproteinase with thrombospondin motifs-4
SD: Standard deviation
ARGS agcan: ARGS neoepitope of aggrecan
IGD: Interglobular domain
SF: Synovial fluid
