# Supplementary material for: Higher aggrecan 1-F21 epitope concentration in synovial fluid early after anterior cruciate ligament injury is associated with worse knee cartilage quality assessed by gadolinium enhanced magnetic resonance imaging 20 years later
Source: BMC Musculoskelet Disord. 2020 Dec 1;21:798. doi: 10.1186/s12891-020-03819-9 (PMC7709245; doi:10.1186/s12891-020-03819-9)
Supplement: Supplementary file 1 — Additional file 1: Supplementary Table S1: Crude linear regression analyses between molecular biomarkers and dGEMRIC, [file 12891_2020_3819_MOESM1_ESM.pdf]

Supplementary Table S1. Crude linear regression analyses between molecular biomarkers and dGEMRIC.

	**Explanatory variables: Acute synovial fluid biomarkers**			**Explanatory variables: Chronic synovial fluid biomarkers**		
	**Dependent variable: dGEMRIC medial**			**Dependent variable: dGEMRIC medial**		
Biomarker	*n*	*Standardized effect*	*95% CI*	*n*	*Standardized effect*	*95% CI*
sGAG	16	-0.211	-0.772 to 0.349	21	0.320	-0.135 to 0.775
1-F21 aggrecan	16	**-0.821**	**-1.148 to -0.494**	14	0.042	-0.586 to 0.671
ARGS aggrecan	18	-0.375	-0.867 to 0.116	22	0.175	-0.284 to 0.634
COMP	13	-0.281	-0.918 to 0.356	12	-0.079	-0.781 to 0.624
MMP-3	18	-0.089	-0.617 to 0.439	12	0.307	-0.364 to 0.978
TIMP-1	18	0.182	-0.339 to 0.703	17	-0.166	-0.709 to 0.376
sGAG/COMP	11	-0.121	-0.869 to 0.628	12	0.294	-0.379 to 0.968
1-F21 aggrecan/COMP	11	**-0.838**	**-1.249 to -0.426**	9	0.098	-0.791 to 0.987
ARGS aggrecan/COMP	12	-0.208	-0.897 to 0.481	12	-0.282	-0.958 to 0.394
MMP-3/TIMP-1	18	-0.294	-0.801 to 0.212	17	-0.195	-0.734 to 0.345

	**Dependent variable: dGEMRIC lateral**			**Dependent variable: dGEMRIC lateral**		
Biomarker	*n*	*Standardized effect*	*95% CI*	*n*	*Standardized effect*	*95% CI*
sGAG	16	-0.188	-0.751 to 0.375	21	**0.453**	**0.025 to 0.881**
1-F21 aggrecan	16	**-0.599**	**-1.058 to -0.141**	14	0.097	-0.529 to 0.723
ARGS aggrecan	18	-0.205	-0.723 to 0.314	22	-0.133	-0.595 to 0.330
COMP	13	-0.003	-0.667 to 0.661	12	0.350	-0.310 to 1.010
MMP-3	18	-0.174	-0.696 to 0.348	12	-0.002	-0.706 to 0.703
TIMP-1	18	0.216	-0.302 to 0.733	17	-0.131	-0.676 to 0.415
sGAG/COMP	11	-0.231	-0.964 to 0.503	12	0.330	-0.335 to 0.995
1-F21 aggrecan/COMP	11	**-0.895**	**-1.231 to -0.559**	9	-0.057	-0.949 to 0.835
ARGS aggrecan/COMP	12	-0.285	-0.961 to 0.390	12	-0.409	-1.052 to 0.234
MMP-3/TIMP-1	18	-0.275	-0.784 to 0.235	17	-0.372	-0.883 to 0.139

	**Dependent variable: dGEMRIC medial + lateral**			**Dependent variable: dGEMRIC medial + lateral**		
Biomarker	*n*	*Standardized effect*	*95% CI*	*n*	*Standardized effect*	*95% CI*
sGAG	16	-0.223	-0.782 to 0.336	21	**0.462**	**0.036 to 0.888**
1-F21 aggrecan	16	**-0.771**	**-1.136 to -0.405**	14	0.080	-0.547 to 0.707
ARGS aggrecan	18	-0.301	-0.806 to 0.205	22	-0.015	-0.482 to 0.451
COMP	13	-0.123	-0.782 to 0.535	12	0.229	-0.456 to 0.915
MMP-3	18	-0.159	-0.682 to 0.364	12	0.113	-0.587 to 0.813
TIMP-1	18	0.227	-0.289 to 0.743	17	-0.161	-0.704 to 0.383
sGAG/COMP	11	-0.197	-0.936 to 0.542	12	0.353	-0.306 to 1.013
1-F21 aggrecan/COMP	11	**-0.934**	**-1.203 to -0.666**	9	-0.006	-0.900 to 0.888
ARGS aggrecan/COMP	12	-0.270	-0.949 to 0.408	12	-0.407	-1.051 to 0.236
MMP-3/TIMP-1	18	-0.316	-0.818 to 0.187	17	-0.339	-0.857 to 0.179

1-F21 aggrecan = 1-F21 epitope of aggrecan, ARGS aggrecan = ARGS neoepitope of aggrecan, COMP = cartilage oligomeric matrix protein, MMP-3 = matrix metalloproteinase 3, sGAG = sulfated glycosaminoglycans, TIMP-1 = tissue inhibitor of metalloproteinase 1.
