# Supplementary material for: Higher aggrecan 1-F21 epitope concentration in synovial fluid early after anterior cruciate ligament injury is associated with worse knee cartilage quality assessed by gadolinium enhanced magnetic resonance imaging 20 years later
Source: BMC Musculoskelet Disord. 2020 Dec 1;21:798. doi: 10.1186/s12891-020-03819-9 (PMC7709245; doi:10.1186/s12891-020-03819-9)
Supplement: Supplementary file 2 — Additional file 2: Supplementary Figure S1: Correlation between the aggrecan biomarkers 1-F21 aggrecan, sGAG and ARGS-aggrecan [file 12891_2020_3819_MOESM2_ESM.pdf]

Figure S1

(A)

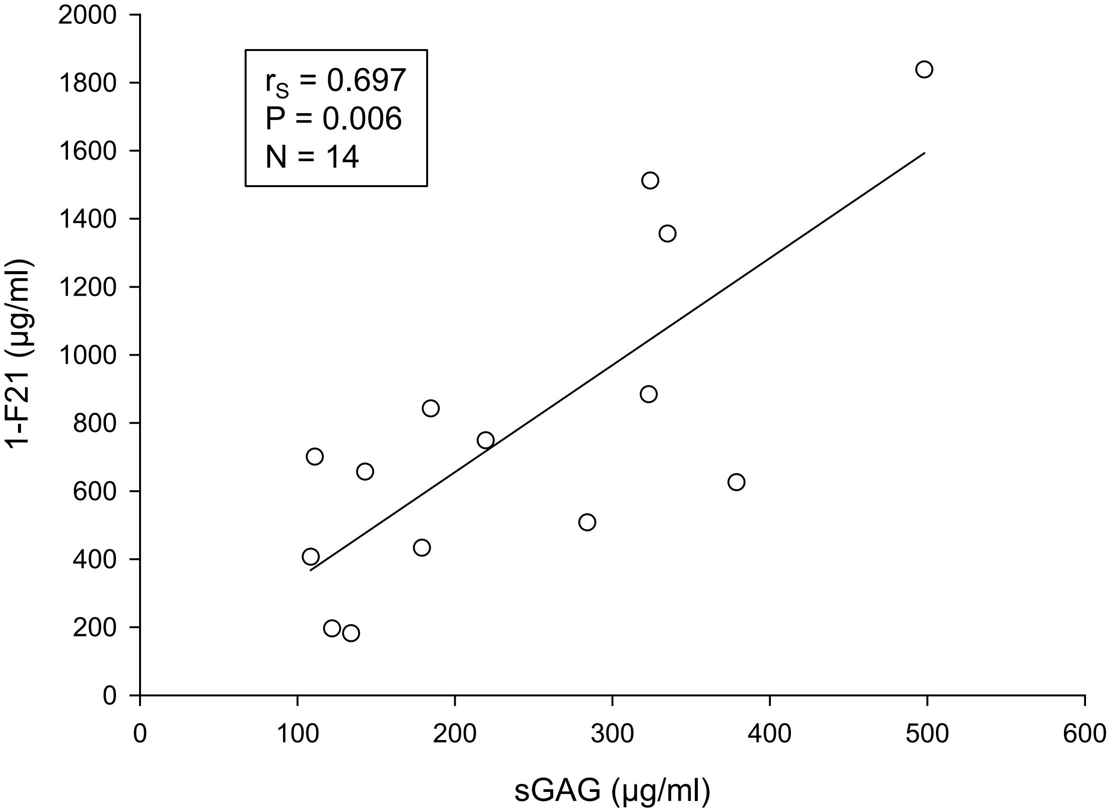

r_S = 0.697
P = 0.006
N = 14
1-F21 (µg/ml)
sGAG (µg/ml)
0
200
400
600
800
1000
1200
1400
1600
1800
2000
0
100
200
300
400
500
600

Figure S1

(B)

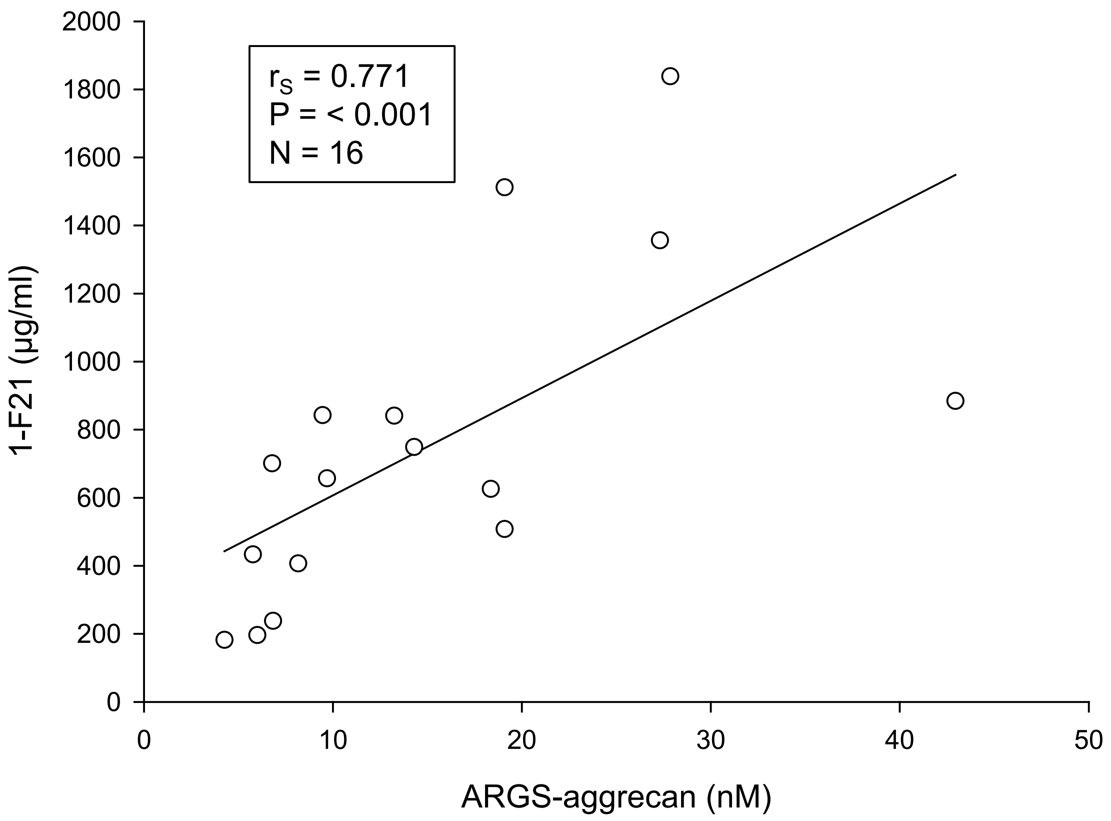

r_S = 0.771
P = < 0.001
N = 16
1-F21 (µg/ml)
ARGS-aggrecan (nM)
0
200
400
600
800
1000
1200
1400
1600
1800
2000
0
10
20
30
40
50

Figure S1

(C)

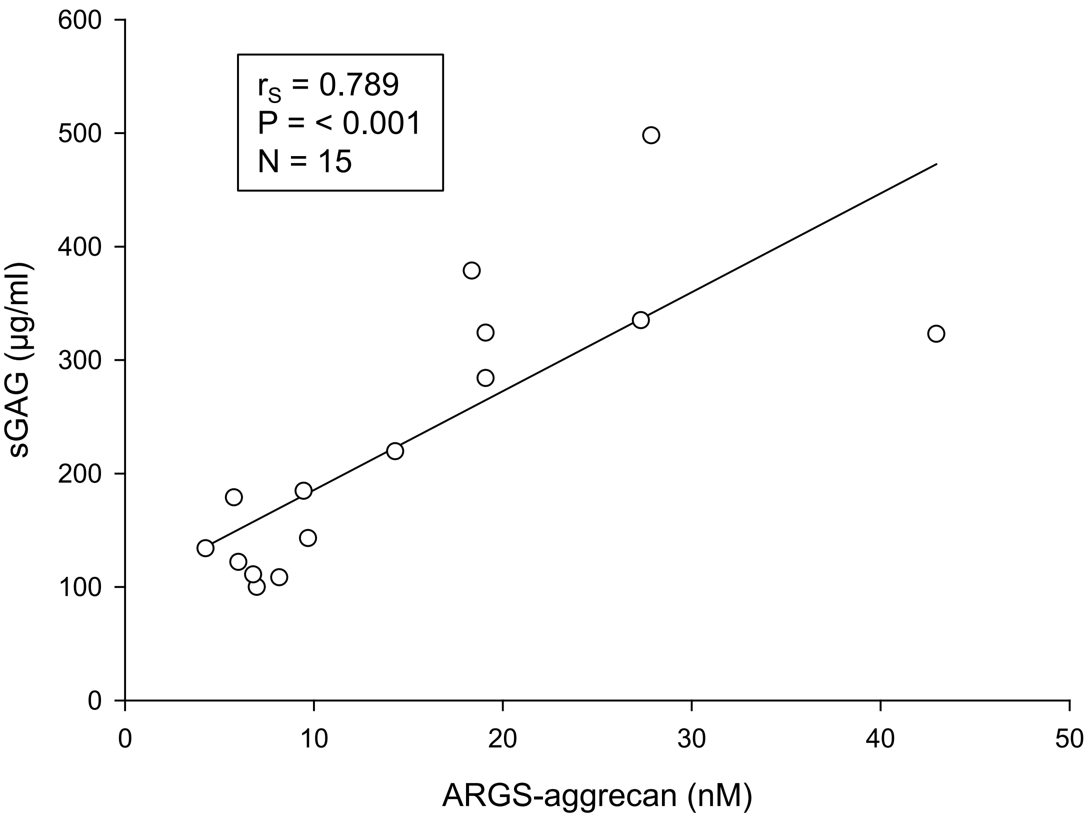

r_S = 0.789
P = < 0.001
N = 15
600
500
400
300
200
100
0
sGAG (µg/ml)
0
10
20
30
40
50
ARGS-aggrecan (nM)

Figure S1. Bi-variate scatter plots between aggrecan markers 1-F21 aggrecan, sGAG and ARGS-aggrecan. Regression lines and correlations, using Spearman's rank correlation coefficient (r_S) and P values, are indicated.
