# Supplementary material for: Higher aggrecan 1-F21 epitope concentration in synovial fluid early after anterior cruciate ligament injury is associated with worse knee cartilage quality assessed by gadolinium enhanced magnetic resonance imaging 20 years later
Source: BMC Musculoskelet Disord. 2020 Dec 1;21:798. doi: 10.1186/s12891-020-03819-9 (PMC7709245; doi:10.1186/s12891-020-03819-9)
Supplement: Supplementary file 3 — Additional file 3: Supplementary Figure S2: Western blot of synovial and cartilage samples; the figure shows uncropped original sized images used in Fig. 3 [file 12891_2020_3819_MOESM3_ESM.pdf]

Figure S2

(Original uncropped membrane images used for Figure 3)

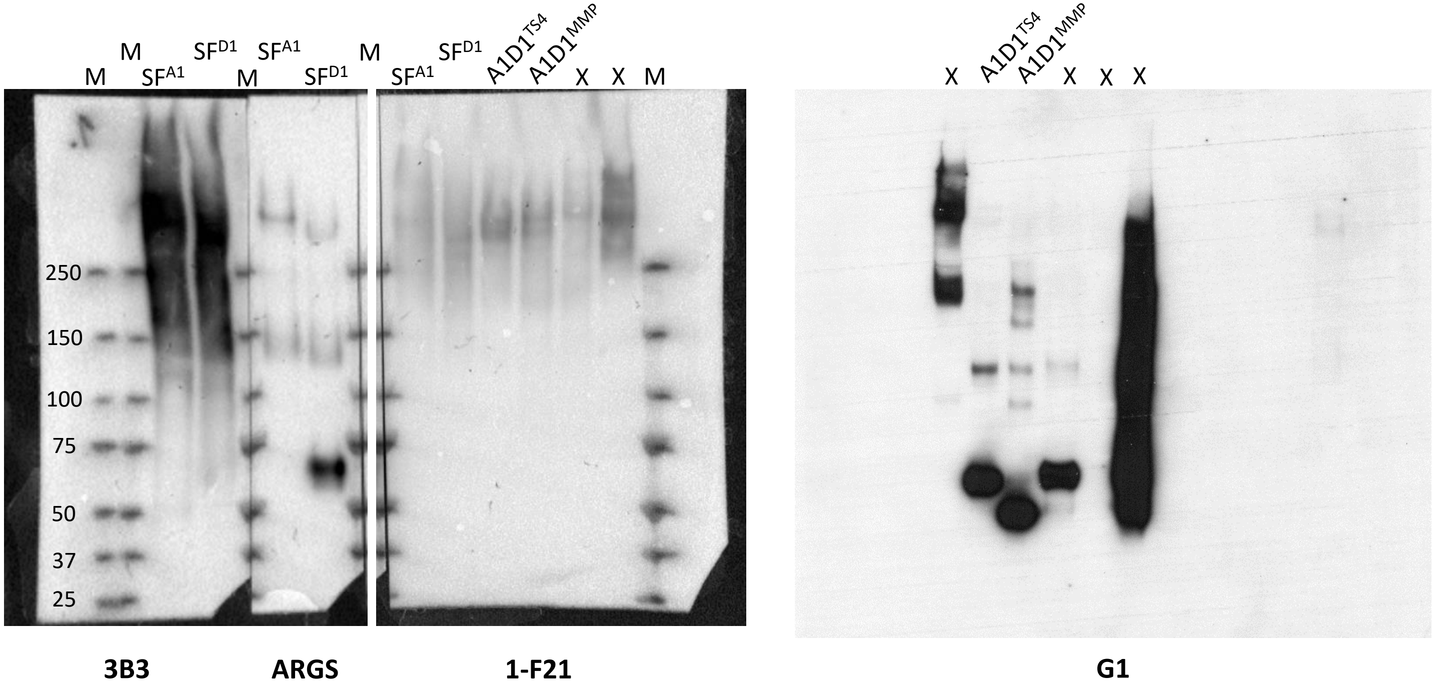

15s exposure
(Bio-Rad ChemiDoc MP)

465s exposure
(Bio-Rad ChemiDoc MP)

60s exposure
(Amersham Hyperfilm ECL)

Figure S2. Western blot of synovial fluid and cartilage samples.
Synovial fluid (SF) A1 and D1 samples on membranes probed with antibodies against 6-sulfated chondroitin sulfate stubs (3B3), aggrecan epitope 1-F21 and ARGS-aggrecan. ADAMTS-4 (TS4) or MMP-3 (MMP) *in vitro* digested cartilage A1D1 aggrecan samples on membranes probed with antibodies against aggrecan epitope 1-F21 and G1-domain of aggrecan. The kDa of molecular weight markers (M) are indicated . The images are from different experiments (with different exposures) showing representative signals from full size blotted gel.
X = samples not discussed (not included) in manuscript.
